# Nanoconfinement drives water ordering

**DOI:** 10.1093/nsr/nwag384

**Published:** 2026-06-22

**Authors:** Si-Ming Wu, Paolo Fornasiero, Xiao-Yu Yang

**Affiliations:** State Key Laboratory of Silicate Materials for Architectures & State Key Laboratory of Advanced Technology for Materials Synthesis and Processing, Wuhan University of Technology, China; Department of Chemical and Pharmaceutical Sciences, University of Trieste and ICCOM-CNR and INSTM Trieste Research Units, Italy; State Key Laboratory of Silicate Materials for Architectures & State Key Laboratory of Advanced Technology for Materials Synthesis and Processing, Wuhan University of Technology, China; Foshan Xianhu Laboratory, China

## Abstract

Direct probing of nanoconfined water reveals that confinement induces water ordering and enables a liquid-solid transition at room temperature, offering a new paradigm for water-dominated reactions.

Confined water is widely present in biological systems, yet its influence on bioactivity and catalytic processes has not been systematically probed [1]. Questions about the key role it plays in these processes are related to more general issues concerning the highly disordered and dynamic nature of the hydrogen-bond network in liquid water, for which it is difficult to construct predictive standardized models. Typically, ordered water structures are stable only at low temperatures (<4°C) or very high pressures (thousands of atmospheres) [2,3], conditions that are not commonly encountered in catalytic and physiological processes. Consequently, ambient-temperature solid-like ordering of water has rarely been considered in conventional models of catalysis and biocatalysis in confined spaces, although earlier simulations predicted that nanoconfinement can stabilize non-bulk ice phases [4]. Recent advances arising from studies by Zheng *et al*. [5] may change this situation. Specifically, the authors have demonstrated that confinement can create ordered water structures under ambient conditions. This finding provides an opportunity to understand the function of confined water and bridge the gap between theoretical models of confined water and real catalytic environments.

It is very difficult to clearly characterize nanoconfined water because it often exists in extremely small amounts buried within solids in a spatially heterogeneous manner. The lack of tools capable of directly probing the structure of water residing inside nanoscale cavities has long hindered systematic investigations and mechanistic analysis. As described in their publication in *Nature Materials*, Zheng *et al*. overcame these challenges using a combination of scanning probe microscopy with nitrogen-vacancy (NV) center–based quantum sensing and engineered nanocavities with precisely controlled heights ranging from sub-nanometer to several nanometers. This approach enabled simultaneous mapping of local confinement geometry and probing of the molecular dynamics and structure of trapped water via nanoscale nuclear magnetic resonance, allowing water behavior to be characterized locally and quantitatively as a function of confinement (Fig. 1).

**Figure 1.**
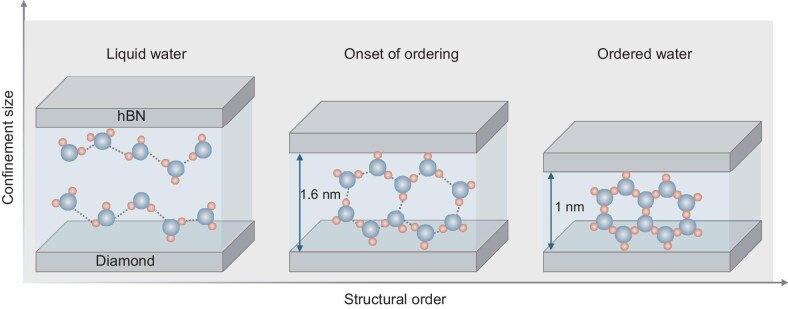
Confinement-driven liquid–solid transition in water. Schematic depiction of water confined between two surfaces with decreasing degrees of separation. While bulk-like confined water (>2 nm) remains liquid, reducing the confinement to 1.6 nm induces partial ordering of the hydrogen-bond network, and further confinement below 1 nm leads to a crystalline arrangement. The figure illustrates how spatial confinement alone can trigger solidification of water at room temperature.

The results of the study conducted by Zheng *et al*. revealed that water confined in >∼2 nm cavities retain liquid-like dynamics at room temperature. When the confinement size is reduced to ∼1.6 nm, molecular motion is markedly suppressed and partial hydrogen-bond ordering emerges. In <∼1 nm cavities, water undergoes complete crystallization. Mechanistically, these transitions can be rationalized by the existence of overlapping interfacial perturbation zones generated by the two confining surfaces. Molecular dynamics simulations show that these zones extend over ∼1.2 nm from the hydrophilic diamond surface and ∼1.8 nm from the hBN surface. Passage through these overlapping zones suppresses the mobility of water and promotes hydrogen-bond ordering. This explains why the water ordering transition starts near ∼1.6 nm, whereas complete crystallization requires confinement below ∼1 nm.

The importance of this work extends far beyond only a demonstration of the existence of a confinement-driven liquid–solid transition of water. Its greatest significance lies in establishing a new framework for modeling water in realistic environments, which reveals the potential role played by confined water as an active catalytic center and provides mechanistic insight into the function of structured water in biological systems and the origin of life.

First, the study by Zheng *et al*. provides quantitative data that help bridge the gap between theoretical descriptions of nanoconfined water and its behavior in realistic environments. By constructing a platform in which confinement geometry can be precisely tuned and locally probed, they showed that disordered liquid water can transform into an ordered solid-like state under sub-nanometer confinement. This shifts the investigation of confined water from indirect inference based mainly on macroscopic observables or simulations toward direct measurements of hydrogen-bond organization and molecular mobility.

Second, the study highlights the potential of confined water as an active catalytic center rather than a passive medium. Zheng *et al*. show that nanoconfinement restricts the motional degrees of freedom of water molecules, decreases entropy, and induces ordering that may influence chemical reactivity. Transitions between disordered and structured hydrogen-bond networks can regulate proton transport, stabilize reactive intermediates, and affect reaction selectivity [6,7]. Notably, recent studies have revealed that confined water can be electrically active and have illustrated the catalytic relevance of confined water by showing that urea can be formed spontaneously from carbon dioxide and ammonia in aqueous droplets [8,9]. In addition, water confined within hydrophobic cavities can undergo a supercritical transition at temperatures lower than that of bulk water [10]. The new perspective challenges conventional enthalpy-focused views of aqueous catalysis by identifying entropy and metastability of water as important contributing variables.

Third, this work also provides insight into physiological water and possible prebiotic chemistry. In living systems, water is rarely present as a bulk liquid; instead, it is confined within membranes, protein cavities, and crowded intracellular environments, where geometry and interfaces continuously reshape its structure and dynamics. The observation that spatial restriction alone can induce water ordering under ambient conditions offers a physical framework for understanding how confined water may participate in molecular recognition, energy transduction, and early chemical organization.

Overall, the observations reported by Zheng *et al*. suggest that nanoconfinement is a powerful means to regulate the structure of water under ambient conditions. This finding could guide the design of catalytic, biological, and materials interfaces in which confined water acts as an active component influencing transport, reactivity, and molecular organization. One important goal of future research in this area would be to establish quantitative relationships that exist between confinement geometry, hydrogen-bond ordering, molecular mobility, and functional performance. These relationships would enable confined water to be treated as a tunable design parameter. Extending this approach to chemically diverse interfaces, asymmetric wettability, ionic environments, and operando conditions could clarify the generality of this ordering mechanism in realistic aqueous systems. Beyond functional materials, this effort also provides a physical framework for understanding how structured water may emerge in prebiotic and biological environments, and how confinement might serve as a fundamental principle by which order arises from disorder.
